# Avacopan as a Steroid-Sparing Therapy in Relapsing Granulomatosis With Polyangiitis

**DOI:** 10.7759/cureus.79072

**Published:** 2025-02-15

**Authors:** Nisha Sapkota, Yubraj Aryal, Prasansa Basnet

**Affiliations:** 1 Medicine, One Brooklyn Health-Interfaith Medical Center, New York, USA; 2 Hospital Medicine, Geisinger Medical Center, Danville, USA; 3 Internal Medicine, Kathmandu University, Biratnagar, NPL

**Keywords:** avacopan, granulomatosis with polyangiitis (gpa), necrotizing and crescentic glomerulonephritis, necrotizing glomerulonephritis, wegener’s granulomatosis

## Abstract

Granulomatosis with polyangiitis (GPA) is a rare autoimmune disease that causes inflammation in small and medium-sized blood vessels, affecting organs such as the lungs, kidneys, and sinuses. We report the case of a 66-year-old man with relapsing GPA. He first presented with sinus and ear symptoms, which were managed with steroids and other immunosuppressive drugs. After many years of remission, he relapsed with nasal congestion, nosebleeds, and lung nodules. During his latest flare, he was treated with high-dose prednisone, rituximab, and avacopan, a new oral drug that blocks the C5a receptor. Avacopan helped reduce inflammation and allowed for a significant decrease in steroid use. The patient’s rapid improvement supports the role of avacopan as a steroid-sparing agent in GPA management, offering a promising way to reduce the harmful side effects of long-term steroid therapy.

## Introduction

Granulomatosis with polyangiitis (GPA), formerly known as Wegener’s granulomatosis, is a type of vasculitis that causes inflammation in small to medium-sized blood vessels [1,2]. It is part of a group of diseases called anti-neutrophil cytoplasmic antibody (ANCA)-associated vasculitides (AAV), which also includes microscopic polyangiitis (MPA) and eosinophilic granulomatosis with polyangiitis (EGPA), also known as Churg-Strauss syndrome [2]. Genetic factors and infections may contribute to GPA, with ANCA targeting proteinase 3 (PR3) in neutrophils, leading to inflammation and blood vessel damage [1,3]. Standard treatment includes high-dose corticosteroids with immunosuppressive drugs like cyclophosphamide or rituximab. However, long-term steroid use can cause serious side effects. Avacopan, a selective C5a receptor inhibitor, is a newer treatment option that helps reduce the need for steroids. In this report, we present a case of relapsing GPA in a 66-year-old male to highlight how avacopan, when combined with rituximab, can be an effective steroid-sparing option to improve the long-term safety and treatment outcomes of GPA.

## Case presentation

**Figure 1:**
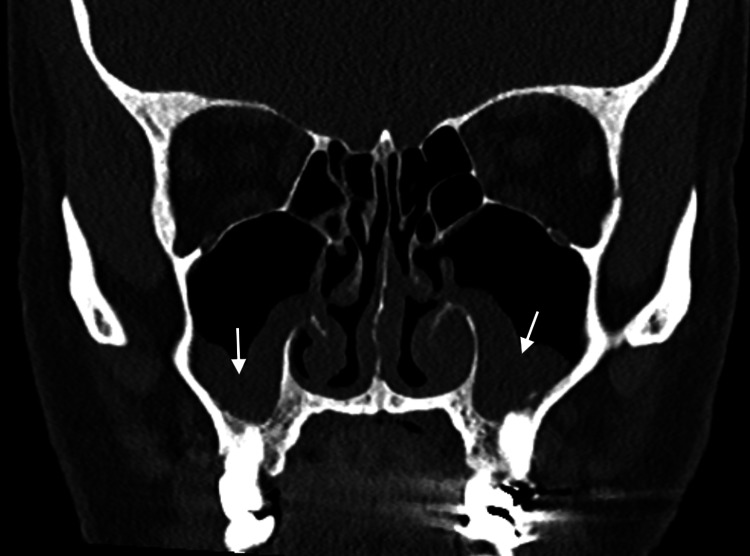
Moderate polypoid mucosal thickening within the bilateral maxillary sinuses. (White arrows) without evidence of bony remodeling.

A 66-year-old male with a past medical history of hypertension and type 2 diabetes presented to the otorhinolaryngology clinic in 2002 with a one-year history of recurrent sinus and ear symptoms, including persistent ear pain, discharge, sinus pressure, facial pain, nasal crusting, and epistaxis. His symptoms were initially managed with multiple courses of antibiotics, nasal steroids, and repeated tympanostomy tube placement, which only provided temporary relief before recurrence. Further evaluation revealed mild normocytic anemia and elevated inflammatory markers. Computed Tomography (CT) of the sinuses demonstrated significant mucosal thickening, suggesting chronic sinusitis (Figure 1).

Urinalysis was unremarkable, and urine sediment analysis showed no dysmorphic red blood cells or casts. A serologic testing evaluation revealed proteinase 3-specific antineutrophil cytoplasmic antibody (PR3-ANCA) positivity with ANCA titer 109.09 (Normal <20.1 units). Initially, there was no lung or renal involvement, and the patient was diagnosed with limited GPA. He was initially treated with prednisone and methotrexate.

**Figure 2:**
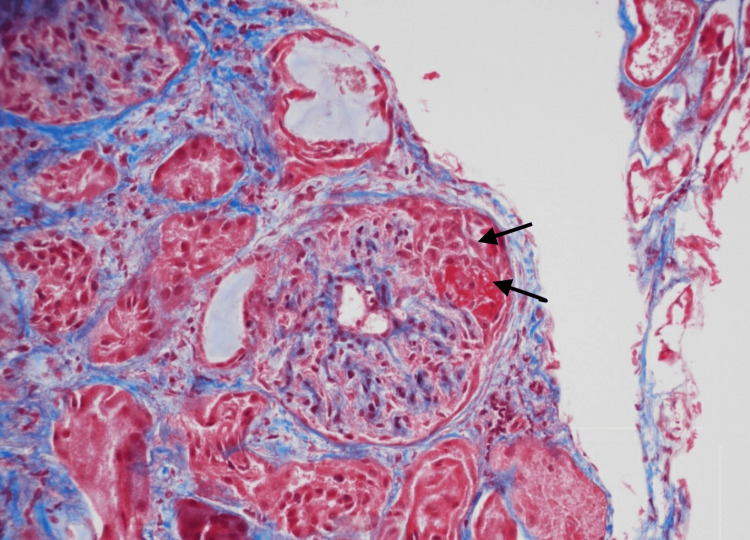
Renal biopsy. Renal biopsy is consistent with necrotizing crescentic glomerulonephritis, pauci-immune type (black arrows indicate segmental fibrinoid necrosis).

However, the patient later developed hematuria and worsening renal function, prompting additional evaluation. Repeat urinalysis revealed microscopic hematuria with dysmorphic red blood cells and red blood cell casts, suggesting glomerular involvement. Serum creatinine was elevated, and a kidney biopsy confirmed necrotizing crescentic glomerulonephritis, pauci-immune type (Figure 2). With this new finding, the diagnosis was revised to systemic GPA. Methotrexate was discontinued, and treatment was switched to steroids and oral cyclophosphamide. Over several months, he showed significant clinical improvement with the resolution of epistaxis and ear symptoms. Hematuria was resolved, renal function was normalized to baseline, and PR3-ANCA titers were gradually downtrended. He transitioned to azathioprine for maintenance therapy, but this was later discontinued following a hospitalization for pancreatitis. Mycophenolate mofetil (CellCept®) was trialed but discontinued due to intolerances, including jitteriness and insomnia. As a result, methotrexate 10 mg weekly was reintroduced.

The patient remained on methotrexate and prednisone for a couple of years before discontinuing all immunosuppressive therapies in 2012. He remained in remission for over a decade, experiencing only intermittent nasal congestion and mild cough but notably no epistaxis, hematuria, or systemic symptoms. His PR-3 ANCA titers remained persistently positive but gradually declined (titer 9.26). Urine analysis continued to show microscopic hematuria, but no acute flare was noted. 

**Figure 3:**
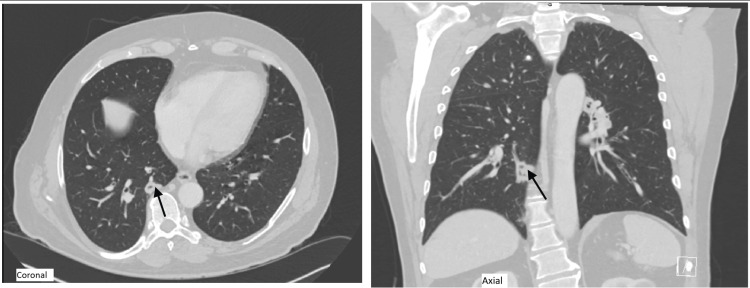
Coronal and axial imaging of the chest. Both images show a cavitary pulmonary nodule in the superior segment of the right lower lobe (shown by black arrows)

After 12 years of remission, the patient relapsed, presenting with refractory sinusitis, epistaxis, nasal crusting, gum sensitivity, cough, and dyspnea. PR-3 ANCA titer rose significantly to 191.1, and ESR and CRP were markedly elevated. Chest CT showed cavitary pulmonary nodules (Figure 3), consistent with GPA recurrence. He was started on induction therapy with rituximab (375 mg/m2 once weekly for four doses) along with a high-dose steroid. Additionally, he was started on avocapon (30 mg twice daily) as a steroid-sparing agent. The steroid was gradually tapered and eventually discontinued. The patient received a rituximab maintenance dose of 1 gm after 6 months and continued avocapon as part of his long-term treatment strategy.

At his one-year follow-up post-relapse, he remained clinically stable, with minimal nasal crusting and resolution of dyspnea and cough. PR3 ANCA titers decreased to 26.1. Repeat imaging of the chest showed no further cavitary pulmonary lesions. His maintenance treatment plan included avacopan and rituximab maintenance therapy (500 mg every six months) to prevent future relapses. 

## Discussion

Granulomatosis with polyangiitis (GPA), formerly known as Wegener's granulomatosis (WG), was first documented in 1933 [1]. GPA is strongly associated with cytoplasmic anti-neutrophil cytoplasmic antibodies (C-ANCA), which are autoantibodies directed against proteinase 3, an enzyme found in the azurophilic granules of neutrophils [3]. Although their presence strongly suggests the disease, a definitive tissue diagnosis remains essential and should not be replaced solely by antibody detection [1,4]. GPA can occur in all racial groups but is more commonly observed in Caucasians. Both men and women are equally affected, with the disease manifesting across a broad age spectrum, from 8 to 99 years old [5].

In the early stages of the disease, patients often present with unexplained constitutional symptoms such as fever and weight loss, which tend to worsen as the disease progresses [4]. The most common initial manifestation is upper airway disease, including sinusitis, oral lesions (ulcers and gingivitis), otitis media, hearing loss, epistaxis, and saddle nose deformity [1,4]. Sinusitis, in particular, is noted in about half to two-thirds of patients with GPA [1,6]. Pulmonary involvement is also a hallmark of GPA, occurring in 45% of patients initially and eventually in up to 87%. Symptoms include cough, hemoptysis, and pleuritis, as well as radiographic findings of pulmonary infiltrates and nodules [4,6]. Renal disease can present early or later, with histological findings ranging from focal segmental to fulminant crescentic glomerulonephritis, potentially leading to chronic renal failure [2,7]. Ocular manifestations, which occur in 29% to 79% of patients, can affect any part of the eye, including keratitis, conjunctivitis, scleritis, episcleritis, uveitis, and more, necessitating comprehensive ophthalmologic evaluation [1,8,9]. Neurological involvement may result in seizures, cerebrovascular accidents, multiple cranial nerve palsies, or mononeuritis multiplex [10]. Additionally, GPA can affect the salivary glands, skin, gastrointestinal tract, and heart.

The diagnosis of GPA is challenging and often delayed due to the wide range of clinical presentations [10]. As a laboratory marker, ANCA is essential for diagnosis, with 82 to 94 percent of GPA and MPA patients testing positive [11]. GPA is primarily associated with PR3-ANCA, while MPA is linked to MPO-ANCA [12]. Other laboratory findings, such as leukocytosis, anemia, and thrombocytosis, are typically nonspecific. Both the erythrocyte sedimentation rate (ESR) and C-reactive protein (CRP) may be elevated in GPA patients and can serve as markers of disease activity. Immunoglobulin levels, especially IgE, may also be elevated [10]. Patients with pulmonary symptoms suspected of having GPA should undergo both a chest radiograph and a CT scan. CT scans can uncover lesions not visible on plain radiographs, such as nodules (especially behind the diaphragm), cavitary nodules, alveolar opacities, and pleural-based lesions [13]. Whenever possible, GPA should be confirmed by biopsy from a suspected active disease site. Tissue samples are usually taken from the skin or kidney, though a thoracoscopic lung biopsy can be an alternative [10]. The biopsy typically shows necrotizing granulomatous inflammation in the respiratory tract, necrotizing vasculitis affecting small-to-medium vessels, and often pauci-immune necrotizing glomerulonephritis [10,14].

Patients should be treated at specialized centers using a multidisciplinary approach. Without treatment, GPA has a very poor prognosis, with an average survival of only five months for untreated patients [1,15]. Medical management focuses on two main goals: inducing remission and maintaining remission. The choice of therapies depends on the stage of the disease [1]. Early induction of remission is essential to reverse inflammation and prevent renal damage, typically beginning within the first 3 months of symptoms in about 40% of cases. This process generally involves combining corticosteroids with cyclophosphamide or rituximab [1,15]. Rituximab (which causes specific B-cell depletion) and cyclophosphamide (which causes immune cell depletion and carries a concern for infertility in younger patients) have similar efficacy, but rituximab is less toxic and better tolerated, making it the preferred choice for remission induction [1,10]. Complete remission with rituximab and corticosteroids can take several months, so patients should be monitored before being considered non-responders. Remission is marked by the resolution of inflammatory symptoms and markers of disease activity (inflammatory markers, ANCA titers, and proteinuria in the case of renal involvement) [1,15]. For non-severe GPA, characterized by vasculitis without life-threatening manifestations, methotrexate is preferred for remission induction because it is less toxic than cyclophosphamide or rituximab [16]. For active remission in newly diagnosed GPA, therapy depends on disease severity and patient factors. Rituximab is preferred for maintaining remission due to its lower relapse rate, but methotrexate, azathioprine, and mycophenolate mofetil (MMF) are also effective. Maintenance therapy typically lasts 18 to 36 months, depending on clinical needs and patient choice [2,15].

Emerging therapies have begun to address these limitations. Research has revealed new treatment targets for GPA, including the alternative complement pathway, which, when activated, produces complement 5a (C5a). Complement 5a (C5a) promotes inflammation by binding to its receptor, which increases adhesion molecules, vascular permeability, and neutrophil activity. ANCA-stimulated neutrophils release C5a, further amplifying inflammation [17,18]. Eculizumab, a humanized anti-C5 monoclonal antibody, has been reported in some GPA cases but was withdrawn due to its interference with the membrane attack complex, which is important for defending against certain pathogenic bacteria [19]. Avacopan, an orally administered selective C5a receptor inhibitor, does not affect membrane attack complex formation and is used as an adjunctive agent with standard induction therapy to reduce glucocorticoid use [17,20]. Avacopan, when added to the standard of care for ANCA-associated vasculitis, was well tolerated and seemed to enhance the time to remission at the higher study dose [20].

Clinical studies have demonstrated that at a dose of 30 mg twice daily, avacopan accelerates remission, improves renal outcomes, and reduces cumulative organ damage, all while allowing for a substantial reduction or complete elimination of high-dose corticosteroids [20]. This steroid-sparing effect is critical in minimizing glucocorticoid-associated adverse effects and enhancing patient quality of life. The clinical utility of avacopan is exemplified by the presented case. The strategic addition of avacopan at 30 mg twice daily served as a steroid-sparing measure, enabling gradual tapering and eventual discontinuation of corticosteroids [17-20]. At one year post-relapse, the patient achieved clinical stability, resolution of pulmonary lesions, improved respiratory symptoms, and a significant reduction in PR3-ANCA titers, underscoring the beneficial impact of avacopan on both disease control and treatment tolerability.

## Conclusions

Standard induction therapy remains effective in achieving remission, but the risk of relapse and adverse effects from prolonged glucocorticoid use highlight the need for safer, more targeted options. Targeting the alternative complement pathway, with C5a playing a key role in GPA pathogenesis, is one such approach. Avacopan, a selective C5a receptor inhibitor, shows promise in mitigating inflammation while preserving essential immune functions. In our case, the patient, followed for one year, showed improvement, but the data is insufficient to establish long-term efficacy. While Avacopan may be beneficial in relapsing cases, further studies are needed to assess its role in standard induction therapy and to evaluate its long-term safety and effectiveness in GPA management.
